# Supplementary material for: Causal interplay between lactose intolerance and gut microbiota: a combined bidirectional Mendelian randomization and in vivo validation study
Source: Front Nutr. 2026 Jun 1;13:1803337. doi: 10.3389/fnut.2026.1803337 (PMC13265576; doi:10.3389/fnut.2026.1803337)
Supplement: Supplementary file 1 [file Data_Sheet_1.zip › supplementary materials/Forward/leave one out cross validation/ebi-a-GCST90027666.finngen_R12_E4_LACTONAS.pdf]

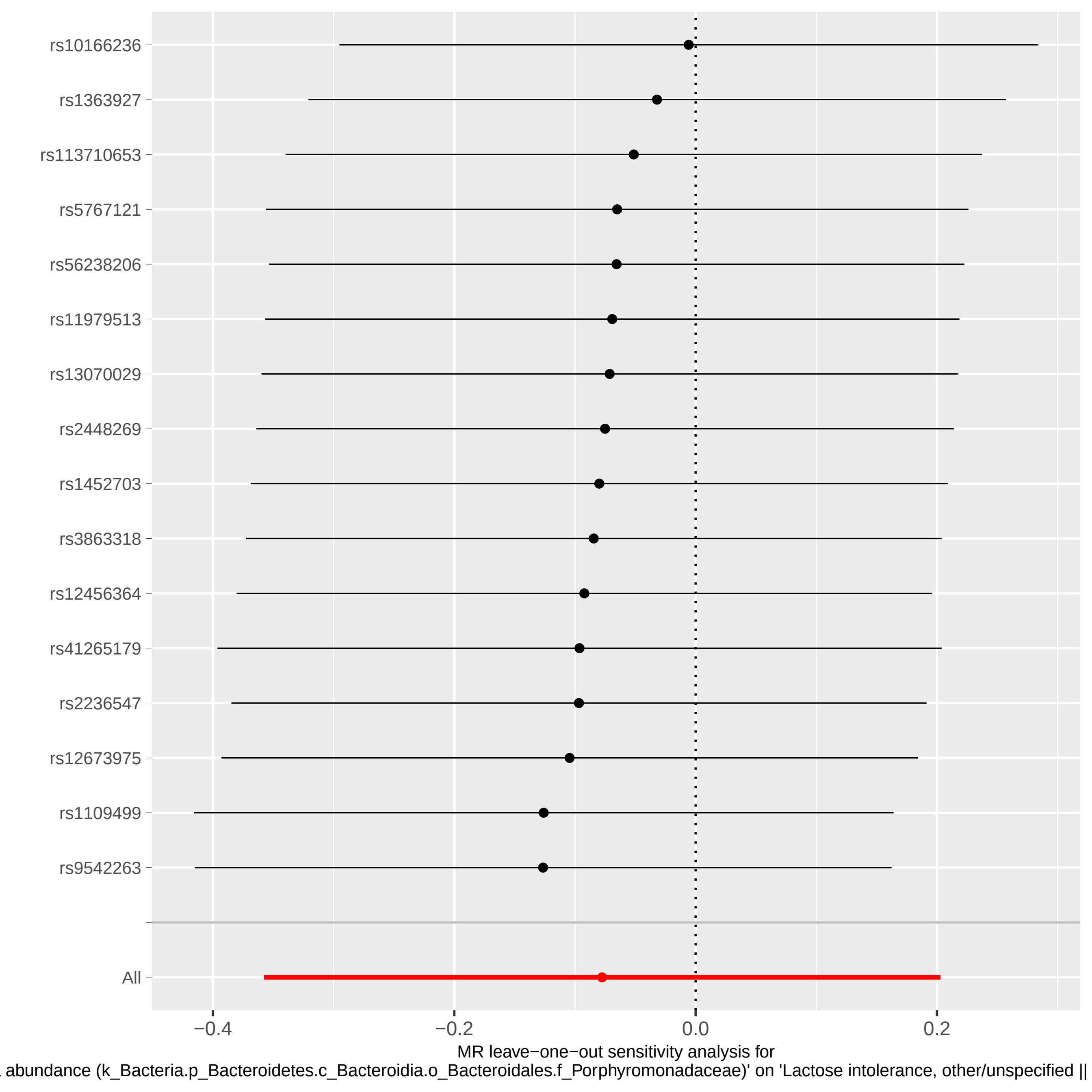
rs10166236
rs1363927
rs113710653
rs5767121
rs56238206
rs11979513
rs13070029
rs2448269
rs1452703
rs3863318
rs12456364
rs41265179
rs2236547
rs12673975
rs1109499
rs9542263
All
−0.4
−0.2
0.0
0.2
MR leave−one−out sensitivity analysis for
abundance (k_Bacteria.p_Bacteroidetes.c_Bacteroidia.o_Bacteroidales.f_Porphyromonadaceae)' on 'Lactose intolerance, other/unspecified ||
